# Conservation of social effects (Ψ*)* between two species of *Drosophila* despite reversal of sexual dimorphism

**DOI:** 10.1002/ece3.3523

**Published:** 2017-10-22

**Authors:** Sarah A. Signor, Mohammad Abbasi, Paul Marjoram, Sergey V. Nuzhdin

**Affiliations:** ^1^ Program in Molecular and Computational Biology Dornsife College of Letters, Arts and Sciences University of Southern California Los Angeles CA USA; ^2^ Graduate Program in Computational Biology Dornsife College of Letters, Arts and Sciences University of Southern California Los Angeles CA USA; ^3^ Department of Preventive Medicine Keck School of Medicine University of Southern California Los Angeles CA USA

**Keywords:** *Drosophila*, indirect genetic effects, locomotion, sexual dimorphism

## Abstract

Indirect genetic effects (IGEs) describe the effect of the genes of social partners on the phenotype of a focal individual. Here, we measure indirect genetic effects using the “coefficient of interaction” (Ψ) to test whether Ψ evolved between *Drosophila melanogaster* and *D. simulans*. We compare Ψ for locomotion between ethanol and nonethanol environments in both species, but only *D. melanogaster* utilizes ethanol ecologically. We find that while sexual dimorphism for locomotion has been reversed in *D. simulans*, there has been no evolution of social effects between these two species. What did evolve was the interaction between genotype‐specific Ψ and the environment, as *D. melanogaster* varies unpredictably between environments and *D. simulans* does not. In this system, this suggests evolutionary lability of sexual dimorphism but a conservation of social effects, which brings forth interesting questions about the role of the social environment in sexual selection.

## 1 INTRODUCTION

Indirect genetic effects (IGEs) occur when individual phenotypes are affected by the genotype of conspecifics with which they interact (Wolf, Brodie, Cheverud, Moore, & Wade, 1998). Essentially, the phenotype of an individual depends on its own genes (direct genetic effects) but also on the genes of its social partners (IGEs). When the social environment contains genes, it can evolve and be an important component of heritability (Bijma, 2014; Dingemanse & Araya‐Ajoy, 2015; Moore, Brodie, & Wolf, 1997). In general, social effects are expected to alter the evolutionary trajectory of other traits such that they evolve differently than expected based on measures of selection and direct additive genetic effects (Wolf et al., 1998). This phenomenon has been demonstrated in a number of species, including laying hens (Brinker, Bijma, Visscher, Rodenburg, & Ellen, 2014; Peeters, Eppink, Ellen, & Visscher, 2012), pigs (Camerlink, Turner, Bijma, & Bolhuis, 2013; Camerlink, Ursinus, Bijma, Kemp, & Bolhuis, 2015), and *Drosophila* (Chenoweth, Rundle, & Blows, 2010; Saltz, 2013; Signor, Abbasi, Marjoram, & Nuzhdin, 2017).

Indirect genetic effects can be described by Ψ, the “coefficient of interaction,” which is the partial regression coefficient of the focal individual's behavior on the behavior of its social partner (Moore et al., 1997). Ψ measures the effect of a trait in the social partner on a trait in a focal individual, such that if it is zero, there is no effect of the social partner on the focal individual. It has been shown previously that Ψ is evolvable (Bleakley & Brodie, 2009; Chenoweth et al., 2010; Kazancıoğlu, Klug, & Alonzo, 2012; Marie‐Orleach et al., 2017). Here, we will focus on the evolution of Ψ measured for locomotion in two species of *Drosophila*.

While few studies have focused on locomotion, it has been found to be an important component of sexual selection and fitness (Ferguson, O'Neill, Audsley, & Isaac, 2015; Husak & Fox, 2008; Lailvaux, Alexander, & Whiting, 2003; Long & Rice, 2007; Perry, 1996; Peterson & Husak, 2006). In *D. melanogaster*, locomotion is sexually antagonistic and is thought to have a shared genetic basis in males and females (Long & Rice, 2007). It is advantageous for males of this species to move more and for females to move less, as higher activity males mate more and higher activity in females’ acts as a stimulus for male courtship (Bateman, 1948; Maklakov & Arnqvist, 2009; Olson‐Manning, Wagner, & Mitchell‐Olds, 2012; Partridge, Green, & Fowler, 1987; Tompkins, Gross, Hall, Gailey, & Siegel, 1982). In *D. melanogaster*, male courtship results in a general reduction in female lifetime reproductive success, likely due to higher rates of remating and interference during oviposition (Bateman, 1948; Fiumera & Dumont, 2006; Kuijper, Stewart, & Rice, 2006; Long & Rice, 2007; Partridge et al., 1987; Tompkins et al., 1982). Sexually antagonistic interactions, and IGEs expressed in those interactions, could potentially have a large impact on the evolutionary trajectory of a given trait (Marie‐Orleach et al., 2017; Moore & Pizzari, 2005).

While the effect of locomotion on fitness has not been explicitly tested in *D. melanogaster*'s sister species *D. simulans*, there is evidence to suggest that locomotion is not sexually antagonistic in this species. As aforementioned, higher activity level in *D. melanogaster* results in increased mating, and multiple matings are detrimental to females and beneficial to males (Fiumera & Dumont, 2006; Kuijper et al., 2006; Long & Rice, 2007; Maklakov & Arnqvist, 2009; Partridge et al., 1987; Tompkins et al., 1982). The relationship between locomotion and mating in *D. simulans* is not known, but there is evidence that undergoing multiple matings is beneficial in *D. simulans* (Bateman, 1948; Fiumera & Dumont, 2006; Kuijper et al., 2006; Maklakov & Arnqvist, 2009; Taylor, Wigmore, Hodgson, Wedell, & Hosken, 2008; Tompkins et al., 1982). While we cannot assume that the relationship between movement and mating is the same, if it were in *D. simulans,* it would suggest that locomotion is not sexually antagonistic. Overall, the data indicate that sexual selection and trade‐offs vary considerably between *Drosophila* species. Indeed, sexual dimorphism of activity level is evolutionarily labile—for example, in *D. suzukii* females are 4× more active than males, while in *D. melanogaster* males are 3× more active than females (Ferguson et al., 2015; Long & Rice, 2007; Signor et al., 2017).

In a previous study, we used measures of Ψ for the effect of male locomotion on female activity to show that there is variation in Ψ between abiotic environments in *D. melanogaster* and found that Ψ was positive. This means that male and female locomotory phenotypes will covary, and females will resemble their male social partners without direct genetic effects (Signor et al., 2017). The two environments were ethanol‐ and nonethanol‐exposed, and while Ψ was higher without ethanol, when Ψ was measured in individual genotypes it varied differently in different genotypes between environments. Here, we will compare Ψ for locomotion in *D. simulans* and *D. melanogaster* in ethanol‐ and nonethanol‐exposed environments to determine whether there has been evolution of social effects between the two species.

Ethanol was chosen as an environmental variable because *D. melanogaster* and *D. simulans* have a different ecological history with regard to ethanol‐rich substrates. *D. melanogaster* is well adapted to ethanol, regularly utilizing resources with ethanol concentrations greater than 8% (Fry, 2014; Gibson, May, & Wilks, 1981; Hoffmann & McKechnie, 1991; Zhu & Fry, 2015). Ethanol tolerance is variable in *D. melanogaster,* and polymorphisms are maintained in the species at multiple loci involved in ethanol metabolism (Chakir, Peridy, Capy, Pla, & David, 1993; Chakraborty & Fry, 2016; Fry, Bahnck, Mikucki, Phadnis, & Slattery, 2004; Fry, Donlon, & Saweikis, 2008). There is a long history of research on the differences in alcohol tolerance between *D. simulans* and *D. melanogaster*, as well as their relative exploitation of this habitat type (David & Bocquet, 1976; Dickinson, Rowan, & Brennan, 1984; McKenzie & McKechnie, 1979; Parsons, 1977). In general, the niche of *D. simulans* is narrower than that of *D. melanogaster*, and it is not found pupating or feeding on high‐ethanol substrates (although larvae may have a higher tolerance) (Chakir et al., 1993; David & Bocquet, 1976; Dickinson et al., 1984; Gibson & Wilks, 1988; Gibson et al., 1981; Joshi & Thompson, 1997; McKenzie & McKechnie, 1979; Mercot, Defaye, Capy, Pla, & David, 1994; Parsons & King, 1977; Parsons & Spence, 1981; Thomson, Jacobson, & Laurie, 1991). Thus, we can investigate the role of social effects in environments to which these two species are, or are not, adapted.

To construct a comprehensive account of Ψ in different environments, we measure movement in *D. simulans* in sets of one female and two males. Across all experiments, female genotype is the same and the male genotype varies (but are identical within a replicate), allowing for genetic replication. We compare these results to those for *D. melanogaster* to investigate how Ψ, and differences in Ψ between environments, has evolved between these two closely related species (Signor et al., 2017). We find that locomotion is remarkably different in *D. melanogaster* and *D. simulans*, with *D. simulans* exhibiting little dimorphism in its level of activity. However, we find that Ψ is almost the same in the two species, indicating that for locomotion sexual dimorphism has evolved, but in this case Ψ did not. This has two implications that Ψ may have a partially separate genetic basis from locomotion in these species and that the differences in Ψ between environments are not due to differences in ethanol tolerance. Lastly, we find that while there is a Ψ × environment interaction in *D. melanogaster,* this is not present in *D. simulans*, which has interesting implications for the maintenance of variation in populations.

## 2 METHODS

### 2.1 *Drosophila simulans* genotypes

Male genotypes were collected from the Zuma organic orchard in Zuma beach, CA in the spring of 2012. They were inbred by 15 generations of full‐sib crosses. A subset of six genotypes were used for behavioral assays. Females used in the behavioral assays were an inbred laboratory strain *y* ^1^ *w* ^1^ (San Diego Species stock number 14021–0251.013).

### 2.2 *Drosophila melanogaster* genotypes

Males were collected from lines originating from an orchard in Winters, California in 1998 that were made isogenic by at least 40 generations of full‐sibling inbreeding (Campo et al., 2013; Yang & Nuzhdin, 2003). A subset of six genotypes were used for behavioral analysis. Female flies were an inbred laboratory strain *y* ^1^ *w* ^1^ (Bloomington stock number 1495).

### 2.3 Heterozygous crosses

Heterozygous crosses were used to generate the male flies used in the behavioral assays. In general, flies collected from the field will be heterozygotes, so in order to more closely replicate these conditions, the genotypes from Zuma Beach and Winters were crossed to a single reference genotype (*D. simulans*:* w* ^501^ San Diego Species stock number 14021–0251.011, *D. melanogaster*:* w* ^1118^, Bloomington stock number 3605), and the F1 flies from this cross were used in the behavioral assays (Nuzhdin, Friesen, & McIntyre, 2012). This allows both the use of heterozygous flies that are more similar to wild flies and the replication of behavioral observations as the flies are genetically identical (Brakefield, 2003; Wahlsten, 2001).

### 2.4 Rearing conditions

All flies were reared on a standard medium at 25°C with a 12‐h light/12‐h dark cycle. Mothers were crossed at 1 day old in groups of 10 males and 10 females. F1 males and females were collected as virgins and maintained separately by sex in vials at a density of 24–30 individuals (males) or 12–15 individuals (females). Males and females were then aged 3–4 days before use in the behavioral assays. To more closely mimic natural situations in which most of the female flies will be mated, 3–5 males of a single standard genotype were added to the virgin females the day before the behavioral observation. There is potential that not all females will have mated, but this effect will be random across genotypes and will not create systematic differences in the data.

### 2.5 Experiment setup

All assays were performed within 2 hr of dawn to avoid any unnecessary variation introduced by circadian rhythms, and replicates were conducted randomly under standardized conditions (25°C, 70% humidity). Each group of two males and one female was put into a single chamber using a paintbrush, for a total of 12 chambers per assay (each chamber is 2.54 cm, VWR cat. no. 89093‐496 see (Signor et al., 2017)). The females were all of the standard *y* ^1^ *w* ^1^ genotypes, and male genotypes varied across replicates but not within them. Each chamber contained 5 ml of either standard grapefruit medium or medium in which 15% of the water has been replaced with ethanol.

To avoid introducing artifacts from the use of CO_2_ for sedation, the flies were sedated at 4°C for 10 min. The flies were allowed to acclimate in the chambers for 10 min before commencing recording with PointGrey Grasshopper digital cameras. This is long enough for any initial startle responses to ethanol to have concluded and is standard for behavioral assays (Cho, Heberlein, & Wolf, 2004; Grosjean et al., 2011; Li, Fink, El‐Kholy, & Roeder, 2015). FlyCapture (PointGrey) was used to properly set up the assays for filming, which was automated using VideoGrabber (http://code.google.com/p/video-grabber). Flies were filmed for 10, 20, or 30 min for three replicates each of the ethanol‐ and nonethanol‐exposed conditions, thus each genotype was filmed in 216 arenas. The different time intervals are intended for an analysis of the transcriptome of flies exposed to ethanol for different time periods, which will be reported in subsequent papers. Thus, for each genotype, three replicates of these 12 chambers were performed for each of three time periods in ethanol‐ and nonethanol‐exposed conditions (12 chambers per replicate, three replicates, three time periods, six genotypes, and two conditions). Arenas with damaged flies or mating events were excluded from the final analysis.

### 2.6 Automatic tracking

In brief, we used a background subtraction method for each frame of the video and a Gaussian mixture model to determine the exact position of each fly. Matching flies in consecutive frames created a representation of fly movement by recording the number of pixels traversed each second by each fly. Please see (Signor et al., 2017) for a more thorough description of the automatic tracking of fly movement. The background subtraction method was modified from that in (Signor et al., 2017), and it is described in the Appendix S1.

### 2.7 Validation of methods

To obtain a measure of error for our tracking algorithms, we randomly selected 360 20‐s intervals from the videos and compared it with the recorded tracking data. We evaluated the frequency of errors using two metrics: (1) how often the tracking switched between two different males (i.e., the fly was incorrectly identified) and (2) how often the sex of the fly was incorrectly inferred.

### 2.8 Movement

The phenotype for each arena was defined as the average movement in pixels per second for each 5‐min interval. For each 5‐min interval, the male phenotype was averaged between the two individuals, as they are of the same genotype. Thus, for each interval, there are two data points, one for females and one for the average of the two males.

### 2.9 Analysis of movement rates and sexual dimorphism

Male and female movements were analyzed separately. We used a t‐test to test for differences between activity levels between species. We used measurements from 779 arenas and normalized the data using a logarithmic transformation. We accounted for repeated measures (the 5‐min intervals for each arena), and fit a linear mixed‐effects model. Random effects included genotype and interactions between genotype, time, and environment. The ID of individual arenas was nested within genotype as a random effect, but it was included with only an intercept term to reflect individual baseline movement but not change over time. Fixed effects were day, environment, time, and the interaction between environment and time. The day of the assay was included to control for any batch effects.

Model fit was evaluated using the lme (nlme) function in R (See Appendix S1 for the R commands). Significance of fixed effect variables was assessed using an F test with the anova (nlme) function. Using the same function, significance of variables of random effect was assessed using a likelihood ratio test to compare model fits with and without the variable of interest. We used REML (restricted maximum likelihood) to fit the lme model parameters. Our fixed effects are common across models, and thus, REML is an appropriate test.

### 2.10 Calculation of Ψ for different abiotic environments

We used the following formula to estimate Ψ:

$$z_{jk}=\alpha +\Psi {\overset{¯}{X}}_{j}+\varepsilon$$

See Table 1 for a description of all of the terms. Ψ is the regression coefficient from this model, which we estimate for each abiotic environment with male phenotype as a fixed effect. It is possible that there is feedback between males and females as well; however, we could not evaluate this with our model. The model was fitted with lme (nlme) in R, and REML was used to fit the lme model parameters.

**Table 1:** A description of the terms used in each model

Symbol	Meaning
*Z*_*jk*_	Phenotype of the female from the *k*th trial with the *j*th male genotype
Ψ	Partial regression coefficient of the focal individual on its social partner
*a*	Effect of female genotype and environment
${\overset{¯}{X}}_{j}$	Mean male movement across all trials containing genotype *j*
*X*_*jk*_	Male movement for the *k*th trial with the *j*th movement
Ψ_*j*_	Ψ including both the effects of shared environment and differences in Ψ due to genotype (*j*)
ε	Error term

### 2.11 Ψ_*j*_ for individual genotypes

To estimate the effect each genotype has on female behavior, we fit a separate regression for each genotype for the effect of male movement on female movement. While Ψ has been reported in the literature for individual genotypes, male and female behaviors can covary for reasons other than Ψ (Bleakley & Brodie, 2009). That is, they can vary due to their shared environment, or due to Ψ*,* and the two factors will always be confounded. We have used extensive measures to control for environment, so if environmental covariation is affecting our estimates, it should be equivalent across experiments. This does not rule out the possibility that there are genetic differences in the sensitivity to shared environment. We calculate it here with the caveat that measures are confounded with shared environment and refer to Ψ as Ψ_*j*_. We used the following formula to estimate Ψ_*j*_:

$$z_{jk}=\alpha +\Psi_{j}X_{jk}+\varepsilon$$

See Table 1 for a description of the variables. The model was fitted as previously described, using lme (nlme) with REML to fit the lme model parameters, accounting for day effect and repeated measures. To estimate Ψ_*j,*_ genotype was included as a random effect, we calculated the slope of the male phenotype effect for each genotype (Appendix S1).

## 3 RESULTS

In the following section, we will include a selection of results from Signor et al. (2017) so that we may explicitly compare between these sister species.

### 3.1 Validation of methods

For *D. simulans,* when flies were far enough from each other to be distinct (~0.5 mm), our annotations were 98% accurate for both measures of error (track switching and sex misidentification). If the flies are in very close proximity, the accuracy of tracking is 5% lower because assigning tracks consistently to adjacent flies is more difficult. However, such errors have little effect on the overall movement rates because the flies occupy the same position. In *D. melanogaster,* our error rates are approximately 1% better in both situations (Signor et al., 2017).

### 3.2 Analysis of movement rates and sexual dimorphism

**Figure 1:**
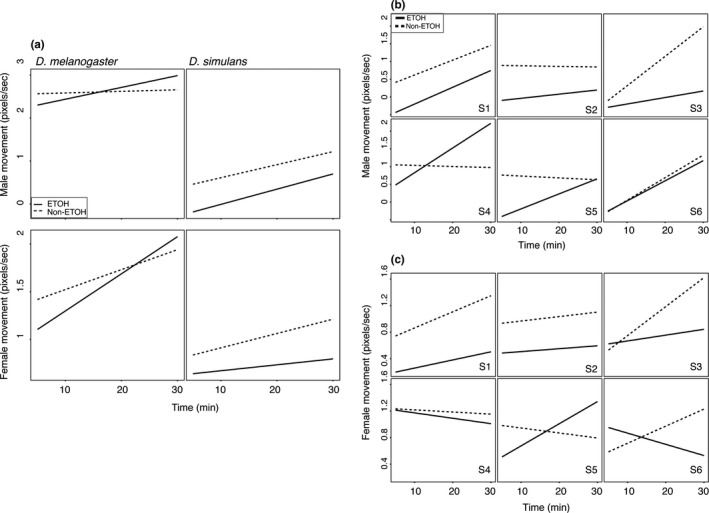
Male and female movements over time in ethanol and nonethanol environments. (a) The log‐transformed average over all male genotypes for movement in *D. melanogaster* and *D. simulans*. The solid and dashed lines represent the linear model fitted to the movement of flies over time in ethanol and nonethanol environments, respectively. (*N* = 1325 measures of movement). (b) Movement broken down by male genotype for *D. simulans*. There is genetic variation in the movement of male flies in different environments (G × E). The *x*‐axis is time in minutes, the *y*‐axis is the log‐transformed movement of male flies in pixels/second. The solid and dashed lines represent the linear model fitted to the movement of flies over time in ethanol and nonethanol environments, respectively. *N* = 240 measures of movement (c) The same results as shown in (b) but for female flies. These females are all the same genotype, and thus, differences in movement will be largely due to male genotype

Overall male movement level in *D. simulans* was lower than in *D. melanogaster*, in both ethanol‐ and nonethanol‐exposed conditions (Figure 1) (Difference in mean movement = 15.23 pixel/s, *t*_1148_ = 41.232, *p *< 10^−4^). However, the effect of genotype and ethanol was significant for male activity level in this species (Figure 1, Table 2) (male movement, genotype, χ^2^ (*df* = 1) = 65.88, *p *< 10^−4^; male movement, environment, *F*_1,705_ = 11.89, *p *=* *.0006)). There was also a much larger decrease in movement in *D. simulans* in response to ethanol than in *D. melanogaster* (Figure 1) (*D. melanogaster* = 10% decrease, *D. simulans* = 42% decrease, *F*_1,1646_ = 61.36, *p *< 10^−4^). In *D. simulans,* we observed a positive slope of movement over time (Table 2) (male movement, time, *F*_1,1870_ = 57.13, *p *< 10^−4^), and the effect was genotype‐specific (Table 2) (male movement, genotype × time, χ^2^ (*df* = 1) = 61.26, *p *< 10^−4^), similar to *D. melanogaster*. Ethanol also affected different genotypes differently (Figure 1, Table 2) (male movement, genotype × environment, χ^2^ (*df* = 1) = 12.95, *p *=* *.0003). However, unlike *D. melanogaster,* we did not observe an effect of ethanol on the slope of movement over time in *D. simulans* (Table 2) (male movement, environment × time, *F*_1,1870_ = 0.32, *p *=* *.57). There was also a three‐way interaction, in that the effect of the environment varied over time between genotypes (Table 2) (male movement, genotype × environment × time, χ^2^ (*df* = 1) = 42.46, *p *=* *.0036). Thus, in both *D. simulans* and *D. melanogaster,* there was an effect of genotype on movement and interactions between genotype, environment, and time.

**Table 2:** Results of the full model for male movement

Fixed effect	*df*	*F*‐value	p‐Value	Random effect	*df*	LRT‐χ^2^	p‐Value
*T*	1	57.13	<10^−4^	*G*_m_	1	65.88	<10^−4^
*E*	1	11.88	.0006	*G*_m_ × *E*	2	12.95	.0003
*E* × *T*	2	0.31	.57	*G*_m_ × *T*	2	61.26	<10^−4^
Day	63	5.47	<10^−4^	*G*_m_ × *E* × *T*	3	42.46	.0036
				Arena	1	599.09	<10^−4^

The variables are time (*T*), environment (*E*), and genotype (*G*). For fixed‐effect variables, the results of the *F* test are shown, and for random‐effect variables, the results of the likelihood ratio test (LRT) to compare model fits are shown.

Female movement was affected by the presence of ethanol (Table 3) (female movement, ethanol: *F*_1,705_ =  8.09, *p *=* *.0046). It was also affected by male genotype; however, there was no interaction between the two terms indicating that the effect of genotype varied in the same way between environments (Table 3) (female movement, male genotype: χ^2^ (*df* = 1) = 16.99, *p *=* *.0019) (female movement, male genotype × environment: χ^2^ (*df* = 1) = 0.013, *p *=* *.91). For female movement, there was, however, an interaction between male genotype and time (Table 3) (female movement, male genotype × time: χ^2^ (*df* = 1) = 16.99, *p *=* *.0002).

**Table 3:** Results of the full model for female movement

Fixed effect	num*df*	den*df*	*F*‐value	p‐Value	Random effect	*df*	LRT‐χ^2^	p‐Value
*T*	1	1870	2.06	.15	*G*_m_	1	16.99	.0019
*E*	1	705	8.09	.0046	*G*_m_ × *E*	2	0.013	.91
*E* × *T*	1	1870	3.19	.075	*G*_m_ × *T*	2	16.99	.0002
Day	67	705	4.85	<10^−4^	*G*_m_ × *E* × *T*	3	3.82	.051
					Arena	1	398.35	<10^−4^

The variables are time (*T*)_,_ environment (*E*), and genotype (*G*). For fixed‐effect variables, the results of the *F* test are shown; for random‐effect variables, the results of the likelihood ratio test (LRT) to compare model fits are shown.

Locomotion in *D. melanogaster* was sexually dimorphic, and we previously confirmed the results of other studies that males move approximately three times more than females (Long & Rice, 2007) (Figure 1a–c). In each species, a single female genotype was used, so any overall variation in movement is due to the abiotic environment and to females’ interaction partners. Sex and genotype are confounded because females are a different genotype than males. We tested the significance of the difference between males and females in *D. simulans* using a linear regression model and found that males moved slightly less than females. This is in contrast with *D. melanogaster*, where males move 2.7× as much as females (0.77× vs 2.7×) (*F*_1,1642_ = 35.29, *p *< 10^−4^).

### 3.3 Calculation of Ψ for different abiotic environments

**Figure 2:**
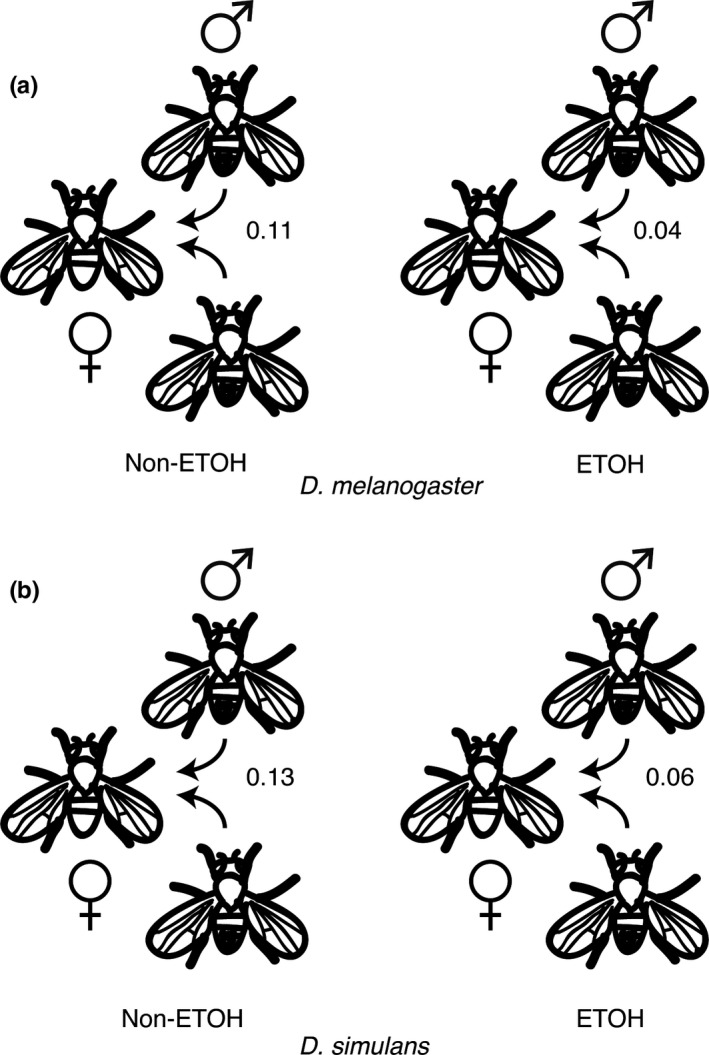
Ψ calculated for each environment in (a) *D. melanogaster* and (b) *D. simulans*. The fitted values were calculated using generalized mixed model (see Methods)

Here, we estimated Ψ in *D. simulans* and compared Ψ between *D. melanogaster* and *D. simulans* (Figure 2a,b). Male phenotype was defined, as before, as the average movement of a male in either ethanol‐ or nonethanol‐exposed conditions. In *D. simulans,* we estimated Ψ* = *0.13 in nonethanol environments and Ψ* = *0.06 in ethanol environments. This is very similar to the estimates of Ψ in *D. melanogaster* Ψ (0.11 and 0.04, respectively) (Figure 2a,b). The authors note that in both cases a single female genotype was used, and it is possible that the inclusion of additional genotypes would alter this comparison.

### 3.4 Ψ_*j*_ for individual genotypes

To determine whether the Ψ is genotype‐specific, and whether that varies with environment, we tested for variation in Ψ_*j*_ (Tables 4 and 5, Appendix S1). With ethanol, we found significant differences between genotypes (genotype × male movement, LRT result for models with and without a genotype × male movement term: χ^2^ (*df* = 1) = 4.95, *p* =.03). This effect is not significant in environments without ethanol (genotype × male movement, LRT results for models with and without a genotype × male movement term: χ^2^ (*df* = 1) = .01, *p* = .91) (Tables 2 and 5). What is unique to *D. simulans* is that they did not vary in unexpected ways between environments, but rather all changed in a consistent manner. That is to say that measures of Ψ_*j*_ were reduced uniformly across genotypes upon exposure to ethanol (genotype × male movement × environment, χ^2^ (*df* = 1) = 0.00002, *p *=* *.99) (Tables 2 and 5). In *D. melanogaster,* Ψ_*j*_ changed differently in different genotypes between environments (genotype × male movement × environment χ^2^ (*df* = 1) = 18.15, *p *< 10^−4^) (Table 4) (Signor et al., 2017).

**Table 4:** Ψ_*j*_ for each genotype in *Drosophila melanogaster*

Genotype	*N*	ETOH	*N*	Non‐ETOH
1	82	0.25	81	0.27
2	101	0.33	81	0.39
3	77	0.31	84	0.16
4	96	0.18	75	0.41
5	79	0.25	80	0.36
6	96	0.19	84	0.54

Shared environment may be conflated with estimates of Ψ_*j*_. This table is reproduced from (Signor et al., 2017).

**Table 5:** Ψ_*j*_ for each genotype in *Drosophila simulans*

Genotype	*N*	ETOH	*N*	Non‐ETOH
1	47	0.29	92	0.79
2	41	0.16	75	0.78
3	38	0.38	100	0.70
4	42	0.38	73	0.83
5	49	0.37	97	0.75
6	47	0.15	78	0.66

Shared environment may be conflated with estimates of Ψ_*j*_.

## 4 DISCUSSION

The role of IGEs and how they vary among environments and species is an important part of understanding the impact of social environments in evolution (Bailey & Zuk, 2012). Few studies have measured IGEs using Ψ for any trait, and this is the only study that compares Ψ for closely related species (Kent, Azanchi, Smith, Formosa, & Levine, 2008; Bleakley & Brodie, 2009; Chenoweth et al., 2010; Bailey & Zuk, 2012; Kazancıoğlu et al., 2012; Bailey & Hoskins, 2014; Bailey et al. 2013). We have demonstrated that despite a reversal in sexual dimorphism for locomotion, Ψ did not evolve between these species. We have shown that for locomotion in *D. simulans* and *D. melanogaster,* Ψ is context‐specific, varying in different environments. However, in *D. melanogaster,* there was a significant interaction with the environment, while in *D. simulans* there was not, indicating that this interaction can evolve between species.

There is a different relationship between male and female locomotions in *D. melanogaster* as compared to *D. simulans*. In *D. melanogaster,* males move 2.7× more than females, while in *D. simulans,* males move only 0.77× as much as females. This difference in sexual dimorphism could have implications for sexual selection in each system given that in *D. melanogaster,* selection on locomotion is sexually antagonistic (Long & Rice, 2007). Absence of sexual dimorphism does not necessarily indicate a lack of sexual conflict, although this has not been investigated in *D. simulans*. Locomotion in other members of this species group has not been well characterized, although there is some evidence that *D. melanogaster* is more active than its close relatives overall (Cobb, Connolly, & Burnet, 1987)*. D. simulans* and *D. melanogaster* do have more divergent courtship behaviors than other members of their species group, likely because they are both cosmopolitan species that occupy many of the same substrates (Cobb, Burnet, & Connolly, 1986; Cobb, Connolly, & Burnet, 1985).

The evolution of locomotory behavior, and of sexual dimorphism in locomotory behavior, has not been well characterized in many systems. The best‐studied systems are lizards and *D. melanogaster*, and in both, locomotion is an important component of sexual selection and fitness (Colomb & Brembs, 2014; Husak & Fox, 2008; Lailvaux et al., 2003; Perry, 1996; Peterson & Husak, 2006). In general, this manifests as males that move faster, but there are exceptions to this that also demonstrate the evolutionary lability of locomotory behavior. For example, in *D. suzukii,* females move 4× more than males, and in one species of lizard movement was not sexually dimorphic despite more active males having higher reproductive success (Ferguson et al., 2015; Peterson & Husak, 2006).

One large difference between selection regimes for females of *D. melanogaster* and *D. simulans* is that in *D. simulans,* multiple matings are beneficial for female fecundity and neutral with respect to longevity, while in *D. melanogaster,* it is neutral to fecundity and deleterious to longevity (Chapman, Liddle, Kalb, & Wolfner, 1995; Kuijper et al., 2006; Taylor et al., 2008; Wigby & Chapman, 2004). Furthermore, other metrics of sexual selection, such as the cost of female choice on longevity or fecundity, indicate that they are largely neutral in *D. simulans* but are costly in *D. melanogaster* (Friberg & Arnqvist, 2003; Pitnick, 1991; Pitnick & Garcia‐Gonzalez, 2002; Taylor, Wedell, & Hosken, 2007; Taylor et al., 2008). While the present work does not measure variation in fitness and therefore cannot make any conclusions about this in relation to locomotion, it may be an interesting question for future research to consider that this may be a manifestation of different selective regimes in the two species. For example, because multiple matings are beneficial to both females and males in *D. simulans*, selection toward a shared level of activity may be beneficial. In contrast, *D. melanogaster* has been selected for higher activity levels because of sexually antagonistic selection (Long & Rice, 2007). This would suggest a lack of sexually antagonistic selection for locomotion in *D. simulans*.

It is interesting that despite these differences in dimorphism and patterns of context‐specific change in locomotion overall, estimated Ψ‐values are approximately equal in *D. melanogaster* and *D. simulans*. This supports two conclusions, (1) that overall differences between abiotic environments for Ψ are not due variation in ethanol tolerance (2) Ψ and locomotion within each species likely have a separate genetic basis, as levels of activity are very different between species and Ψ is not. Thus, the two traits would be able to evolve independently, contributing to sexually antagonistic selection in *D. melanogaster* and sexual selection in *D. simulans*. It is clear that despite a reversal in sexual dimorphism, and different sexual dynamics, Ψ has not evolved between these two species.

In light of the fact that *D. melanogaster* is adapted to substrates with high concentrations of ethanol, while *D. simulans* is not, it is interesting that it is *D. melanogaster* that exhibits a Ψ_*j*_ × environment interaction. There is considerable spatial heterogeneity in the ethanol content of the environment for *Drosophila*, which implies that not all genotypes will encounter ethanol‐rich substrates (Hoffmann & McKechnie, 1991; McKenzie & McKechnie, 1979). Polymorphisms for ethanol tolerance are widespread in *Drosophila* species. It has previously been shown that variable exposure to ethanol in *D. melanogaster* maintains a balanced polymorphism in the *Aldehyde dehydrogenase* gene responsible for detoxifying acetaldehyde derived from dietary ethanol (Chakraborty & Fry, 2016). Furthermore, there is a long history of documenting variation and latitudinal clines in *Alcohol dehydrogenase*, which transforms ethanol into acetaldehyde (Dorado & Barbancho, 1984; Gibson et al., 1981; Mercot et al., 1994; Zhu & Fry, 2015; Ziolo & Parsons, 1982). Thus, it would be interesting to consider that adaptations for Ψ on ethanol substrates could be maintained as polymorphisms in the population, including locomotion. If this were the case, this would not have occurred in *D. simulans* due to its avoidance of substrates containing high concentrations of ethanol. While it is slightly counterintuitive to imagine than a lack of selection maintains less variation in a trait, this is the expectation if spatially variable selection is common, and polymorphisms are conditionally beneficial.

*Drosophila melanogaster* and *D. simulans* are both cosmopolitan species commonly found in the same habitats. *D. simulans* readily evolves increased ethanol tolerance in the laboratory, so it may be that selection for whatever benefit ethanol provides resulted in different trade‐offs in *D. melanogaster* compared to *D. simulans* (Joshi & Thompson, 1997; Lefèvre, de Roode, Kacsoh, & Schlenke, 2012). For example, polymorphisms at the *Aldehyde dehydrogenase* locus in *D. melanogaster* are detrimental in the absence of ethanol as they result in a reduction in the efficacy of processing other targets (Chakraborty & Fry, 2016). However, ethanol‐rich substrates provide some protection against parasites for *D. melanogaster*, while *D. simulans* appears to mount a stronger immune response instead (i.e., fight or flight) (Lefèvre et al., 2012; Milan, Kacsoh, & Schlenke, 2012). The relationship between ethanol, parasite resistance, adult and larval tolerance, and caloric benefit is not entirely clear despite a number of studies on the subject (Chakir et al., 1993; Dorado & Barbancho, 1984; Fry et al., 2004, 2008; Garcin, Cote, Lau You Hin, Chawla, & Radouco‐Thomas, 1986; Hodges, Laskowski, Squadrito, De Luca, & Leips, 2013; Kerver & Rotman, 1987; McKechnie & Morgan, 1982; McKenzie & McKechnie, 1979; Mercot et al., 1994; Milan et al., 2012; Muhammed‐Ali & Burnet, 1995; Thomson et al., 1991).

Attempting to estimate Ψ_*j*_ requires confounding the effect of shared environment and the effect of different genes in the environment. As such it is an overestimation of the effect of individual genotypes, which is most likely why values of Ψ_*j*_ are consistently higher than Ψ. However, there is no other way to attempt to calculate this term, and extensive environmental controls were used such that the effect of shared environment should have been constant across assays. This does not preclude the existence of genetic variability in the response to shared environment in males, and thus, it is possible that even the extensive environmental controls do not eliminate this effect. As such Ψ_*j*_ is a valuable contribution to understanding the patterns in our data, however the authors stress that absolute values are not interpretable and it must be considered with caution.

In conclusion, we have explored differences in Ψ for locomotion between abiotic environments for two closely related species, *D. simulans* and *D. melanogaster*. We have found extensive evidence for context‐specific Ψ that varies between species, with *D. melanogaster* having interaction terms with abiotic environment that are lacking in *D. simulans*. We have found that Ψ is positive in both abiotic environments for both species, and approximately equal within a given abiotic environment between species. We have also found evidence for differences in sexual dimorphism in locomotion despite similarities in Ψ between species. These findings present interesting pathways for future research into the evolution of sexual antagonism and sexual selection, and the role of IGEs in both of these processes.

## CONFLICT OF INTEREST

None declared.

## DATA ACCESSIBILITY

Data deposited in the dryad repository: https://doi.org/10.5061/dryad.p3215 and https://doi.org/datadryad.org/resource/10.5061/dryad.bf278.

## AUTHOR CONTRIBUTIONS

SS performed the experiments, wrote the manuscript, and created the figures. MA analyzed the data. PM and SN conceived of the experiments and contributed to their design.
