# Supplementary material for: Conservation of social effects (Ψ) between two species of Drosophila despite reversal of sexual dimorphism
Source: Ecol Evol. 2017 Oct 22;7(23):10031–41. doi: 10.1002/ece3.3523 (PMC5723616; doi:10.1002/ece3.3523)
Supplement: Supplementary file 1 [file ECE3-7-10031-s001.docx]

**Supplementary Methods**

*Background subtraction in D. simulans*

The background subtraction method used for *D. melanogaster* could not be used for *D. simulans*, as they are less active. The method used for *D. melanogaster* is described in [9]. For *D. simulans* we used a histogram method, which exploits the assumption that a small proportion of pixels in the background image correspond to flies that aren’t moving. Specifically, the lowest 1% quantile for intensity will contain the darker regions of the image, which indicates the position of the flies. To create the image that will be used as the background, these pixels are then replaced with the pixel intensity corresponding to the 1% quantile. This creates a brighter background image to use for subtraction by increasing the contrast between the background and the flies.

This process is repeated for all arenas in our experiment to produce the full background image.

*Movement analysis*

We standardized movement for male and female flies separately, with a mean of 0 and standard deviation of 1. lme(nlme) in R was used to evaluate the effect of different variables on movement. The output is in Table 2 and 3 and the commands are as follows:

*Male movement*

lme (Male Movement ~ Time * Environment + Day, random = list (Genotype ~ 1+Time * Environment, Arena_ID = ~ 1))

*Female movement*

lme (Female Movement ~ Time * Environment + Day, random = list (Genotype ~ 1+Time * Environment, Arena_ID = ~ 1))

Significance of each variable was assessed by comparing model fits using anova(nlme)in R.

*Indirect genetic effects between abiotic environments*

*Ψ* was calculated using the average movement of male flies for each genotype in a given environment (GEMM) as a predictor of the movement phenotype of the female partner. Day and Arena ID were included to allow for repeated measures and account for batch effects. The results of all of these models are shown in Figure 2, and the R code is as follows:

lme (Female Movement ~ Time + Day+GEMM, random = list (Arena_ID = ~ 1 ))

*Effect of ethanol on Ψ*

To determine if there was an effect of ethanol on *Ψ* we tested the significance of the interaction between GEMM and environment*.* This is done using the mixed model defined below, which accounts for the effects of Day and Arena ID. The full output is not included in the manuscript and is shown here in addition to the R code.

lme (Female Movement ~ Time + Day+GEMM*Environment, random = list (Arena_ID = ~ 1) )


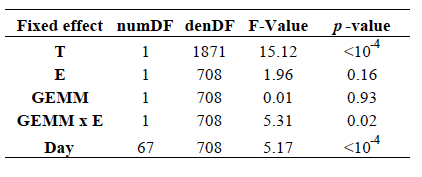


*Ψ_j,_ Regression of individual genotypes*

*Ψ_j_* was estimated by fitting a slope to each genotype for male movement. *Ψ_j_* was calculated separately for each environment.

lme (Female Movement ~ Time + Day+ Male Movement, random = list (Genotype=~ 1+Male Movement, Arena_ID = ~ 1))

We included a random effect of male genotype to allow for the genotype-specific regression terms for male movement to have different intercepts. This allows for differences in baseline female movement rates as a function of male genotype. The output for this model is included in Table 4 and 5.

*Variation in Ψ_j_ between genotypes*

We test the hypothesis that *Ψ_j_*varies between genotypes against the null hypothesis that *Ψ_j_*is constant. The null model does not contain a genotype-specific slope term. We used a likelihood ratio test between the full and null models to assess the significance of the male genotype x movement interaction. The null model is as follows and the results are discussed in the body of the paper under *Ψ_j_* for individual genotypes:

lme (Female Movement ~ Time + Day+ Male Movement, random = list (Genotype=~1, Arena_ID = ~ 1))

*The effect of ethanol on Ψ_j_*

We also wanted to test for the possibility that there is a genotype specific effect of ethanol on movement. This would be a three-way interaction, between genotype x male movement x environment. To test the hypothesis that there is variation in the effect of ethanol on *Ψ_j_* against the null hypothesis that the effect of ethanol on *Ψ_j_*is constant, we use the model defined bellow and test for the significance of the three-way interaction term of male movement x male genotype x environment using a likelihood ratio test. The results of the model are also reported in the paper.

lme (Female Movement ~ Etoh*Time + Day+Etoh* Male Movement, random = list (Genotype=~1+Male movement*Etoh, Arena_ID = ~ 1 ) )

**
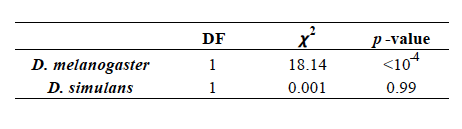
**
